# Clinical Characteristics and Treatment Strategy of Retroperitoneal Schwannoma Adjacent to Important Abdominal Vessels: Three Case Reports and Literature Review

**DOI:** 10.3389/fsurg.2020.605867

**Published:** 2021-01-14

**Authors:** Qi Wu, Bingqiang Liu, Jun Lu, Hong Chang

**Affiliations:** Department of Hepatobiliary Surgery, Provincial Hospital Affiliated to Shandong University, Jinan, China

**Keywords:** retroperitoneal tumor, schwannoma, vascular, clinical characteristics, treatment

## Abstract

**Purpose:** The purpose of this study was to review the clinical characteristics and treatment strategies of patients with retroperitoneal schwannomas adjacent to important abdominal vessels.

**Case Presentation:** A total of three patients with retroperitoneal schwannoma immediately adjacent to important blood vessels in the abdominal cavity underwent successful surgical resection. They all had symptoms of abdominal pain and discomfort, two cases underwent three-dimensional reconstruction. There were no serious complications such as peripheral blood vessels and organ damage in all three cases. One case had chyle leakage after surgery, conservative treatment was successfully discharged.

**Conclusions:** Retroperitoneal schwannomas immediately adjacent to important abdominal vessels have unique clinical characteristics. Preoperative three-dimensional reconstruction can fully show the local vascular relationship of the tumor, which is conducive to surgical planning and risk assessment. Benign tumors with large size and adjacent complex vessels can still be completely resected by surgery. Laparotomy resection is safe and feasible.

## Introduction

Retroperitoneal schwannoma is a rare clinical entity, accounting for only 1% of all retroperitoneal tumors (1). They are soft tissue tumors and originate from Schwann cells in the peripheral nerve capsule. However, when they are closely adjacent to blood vessels, the tumor may originate from the blood vessel wall, to be precise, from the peripheral nerve fibers on the adventitia of the blood vessel wall (2). Therefore, it should be considered tumors that compress blood vessels may themselves originate from blood vessels, because this is critical to the choice of treatment options. They are usually benign, accounting for nearly 8% of retroperitoneal benign tumors (3), and malignancy is rare. Schwannomas usually occur in the peripheral nerves of the head, neck or limbs, only 0.5–5% occur in the retroperitoneum (4), and most of them are located in the paravertebral space or presacral area (5), compared with other parts, they are more prone to spontaneous degeneration and bleeding (5, 6). Due to its location, retroperitoneal schwannoma tends to be closely related to abdominal large blood vessels (5). In the case of complex blood vessels adjacent to it, retroperitoneal schwannoma shows different clinical features, and its complete surgical resection is still difficult and high-risk. Intraoperative side injuries such as massive hemorrhage, damage to nearby organs, and organ ischemia can be fatal. Postoperative neurological dysfunction is a serious complication due to its neural origin. However, the application of three-dimensional reconstruction techniques, intraoperative nerve monitoring techniques (7, 8), and the magnifying effect of laparoscopy (9, 10) may provide help for our surgical resection and good prognosis. We report three cases whose retroperitoneal schwannomas adjacent to important abdominal vessels were completely removed to provide clinicians with experience in diagnosis and treatment.

## Case Presentation

### Case 1

**Figure 1:**
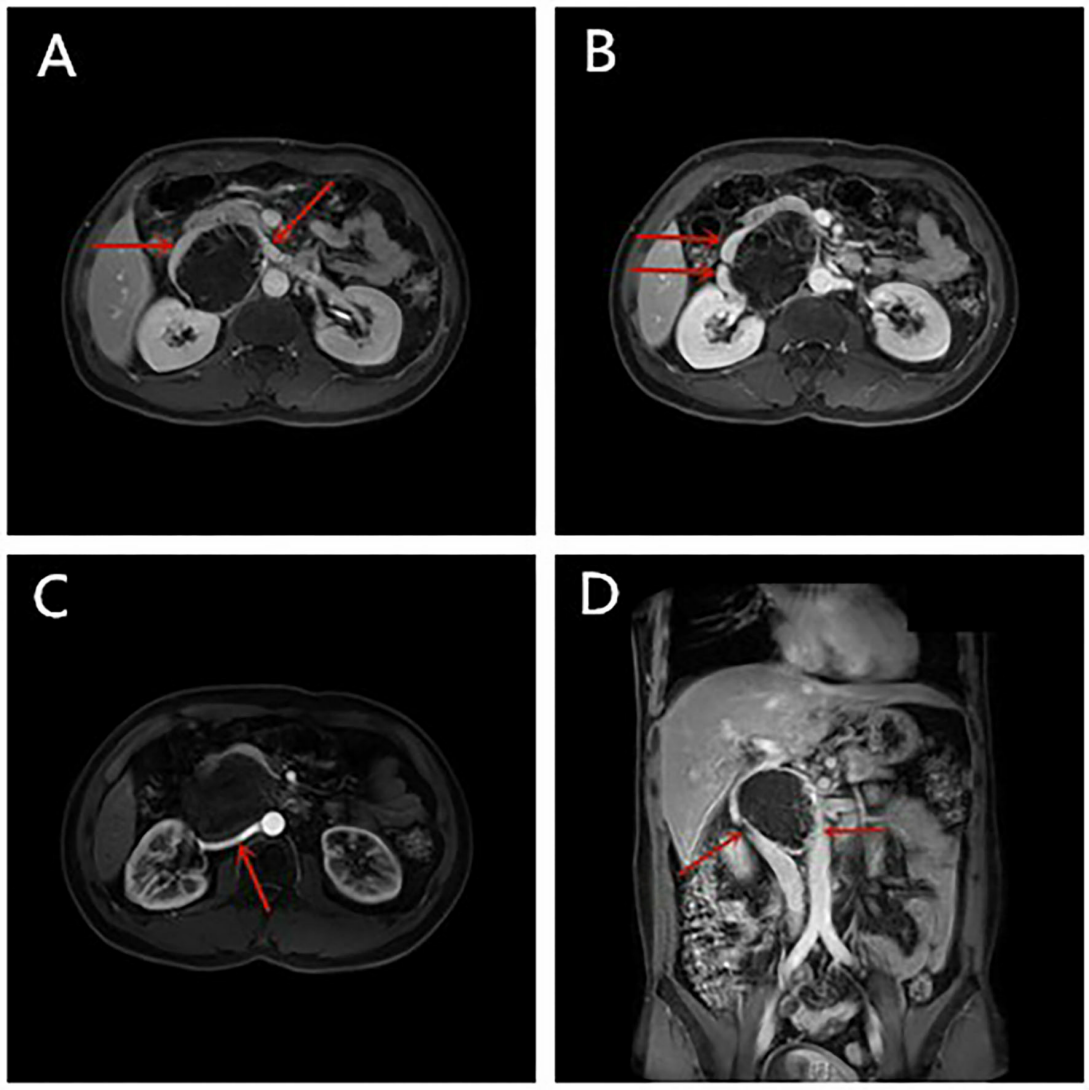
**(A–D)** The enhanced Magnetic Resonance Imaging (MRI) performance of Case 1.

A 40-year-old man suffered from intermittent left abdominal distension and pain for more than 1 month, and Computed Tomography (CT) in a local hospital revealed the possibility of lymphocele. Enhanced Magnetic Resonance Imaging (MRI) showed (Figure 1) with the maximum section of about 8.2 cm × 5.9 cm, the boundary of the mass was unclear. The patient was taken a transabdominal straight muscle incision in the right upper quadrant, and the mass was cystic and solid, with a complete capsule; the upper edge of the tumor raised the vein together with the left renal vein and right renal vein superiorly and laterally, the inner edge of the tumor was adjacent to the duodenum, the lower edge of the tumor was adjacent to the right renal artery and abdominal aorta, and the right renal capsule was squeezed inferiorly outside the tumor. The gastrocolic ligament was opened and the descending duodenum was mobilized with a Kocher incision to fully expose the anteromedial aspect of the tumor. Carefully separated the surrounding blood vessels along with the tumor capsule, completely dissected the tumor, ligated and cut it into the tumor vascular lymphatic tissue. The operation went smoothly and the bleeding was about 100 ml. The postoperative histology showed that combined with the results of immunohistochemistry, it was consistent with schwannoma, accompanied by significant degeneration and cystic degeneration. Immunohistochemical staining was positive for S100, SOX10 and vimentin, negative for Syn, CD34, CD117 and SMA, and ki-67+ (2%).

### Case 2

**Figure 2:**
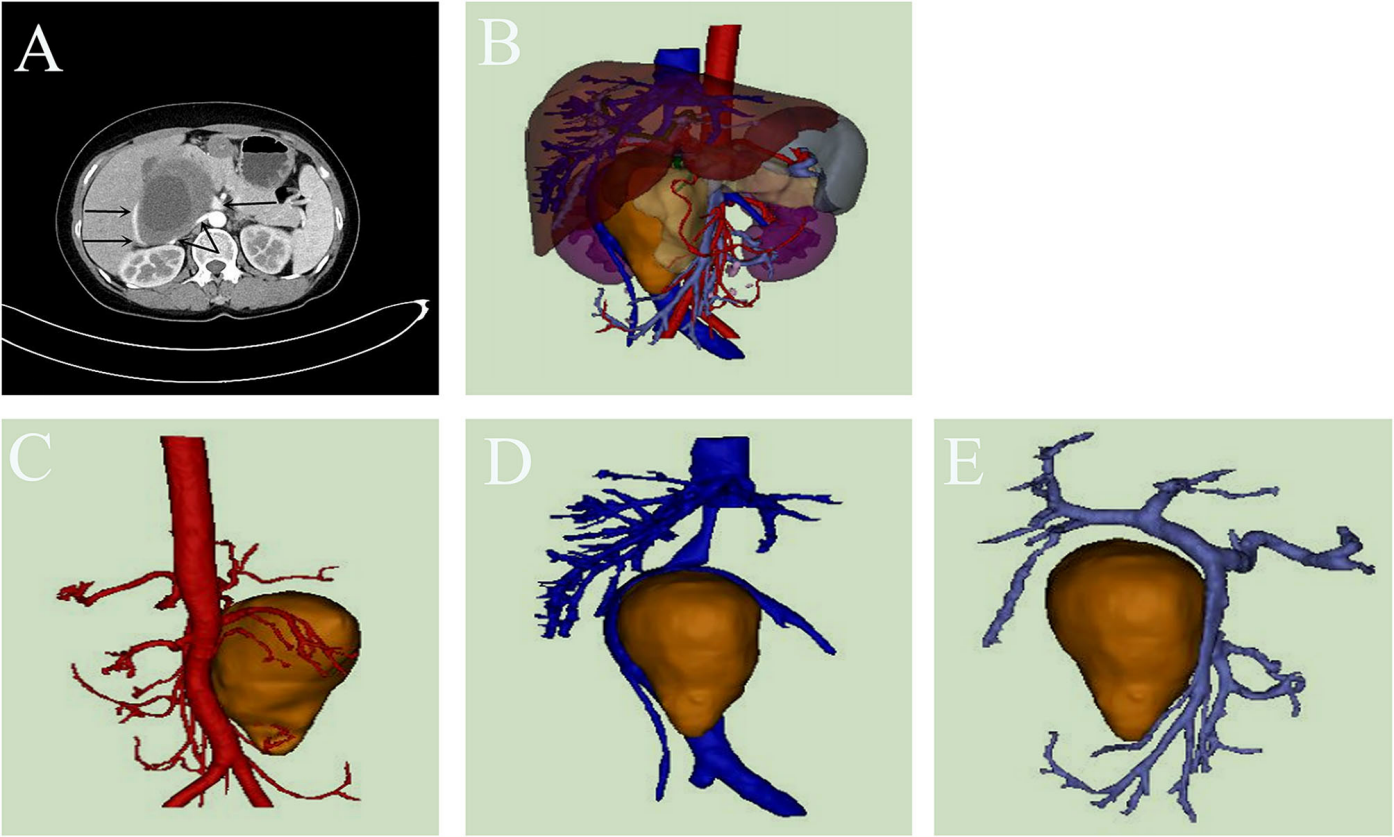
**(A–E)** The enhanced Computed Tomography (CT) and three-dimensional reconstruction performance of Case 2.

A 41-year-old woman had upper abdomen pain and discomfort for a week, which worsened after eating. MRI at the local hospital showed neurogenic tumor or pancreatic tumor. Enhanced CT showed (Figure 2) a retroperitoneal soft tissue mass with a maximum section of about 10.0 cm × 8.7 cm. The three-dimensional reconstruction clearly showed the left renal vein and pancreas in front of the tumor, the left renal vein, right renal artery and abdominal aorta on the left, the inferior vena cava and right renal vein on the right, and the right renal artery and vein and right renal hilum at the back. This patient was taken a right abdominal transabdominal right muscle incision, about 20 cm in length. The tumor was located in the right upper abdomen, posterior to the duodenum, smooth surface, tough texture, relatively complete capsule, clear boundary, the vein was squeezed to the right flexion, the left posterior of the tumor closely adhered to the abdominal aorta, and no lymphadenopathy was observed in the hepatoduodenal ligament. The duodenum was lifted, the posterior peritoneum was opened, the loose space between the duodenum and the tumor was freed, the level of the abdominal aorta was freed along the left side of the tumor, the adhesion between the tumor and the abdominal aorta was bluntly and sharply separated, the active bleeding point was ligated, the tumor was freed from the retroperitoneum, the right renal artery and left and right renal veins were exposed to protect during the operation, the vein was exposed, the space between the tumor and the vein was bluntly and sharply freed, the tumor was completely removed. The operation was uneventful, with intraoperative bleeding of about 100 ml. The postoperative histopathology was consistent with retroperitoneal schwannoma. Immunohistochemical staining was positive for S100 and PGP9.5, negative for NF, CD34, Desmin, CD117 and Dog-1, and ki-67+ (3%).

### Case 3

**Figure 3:**
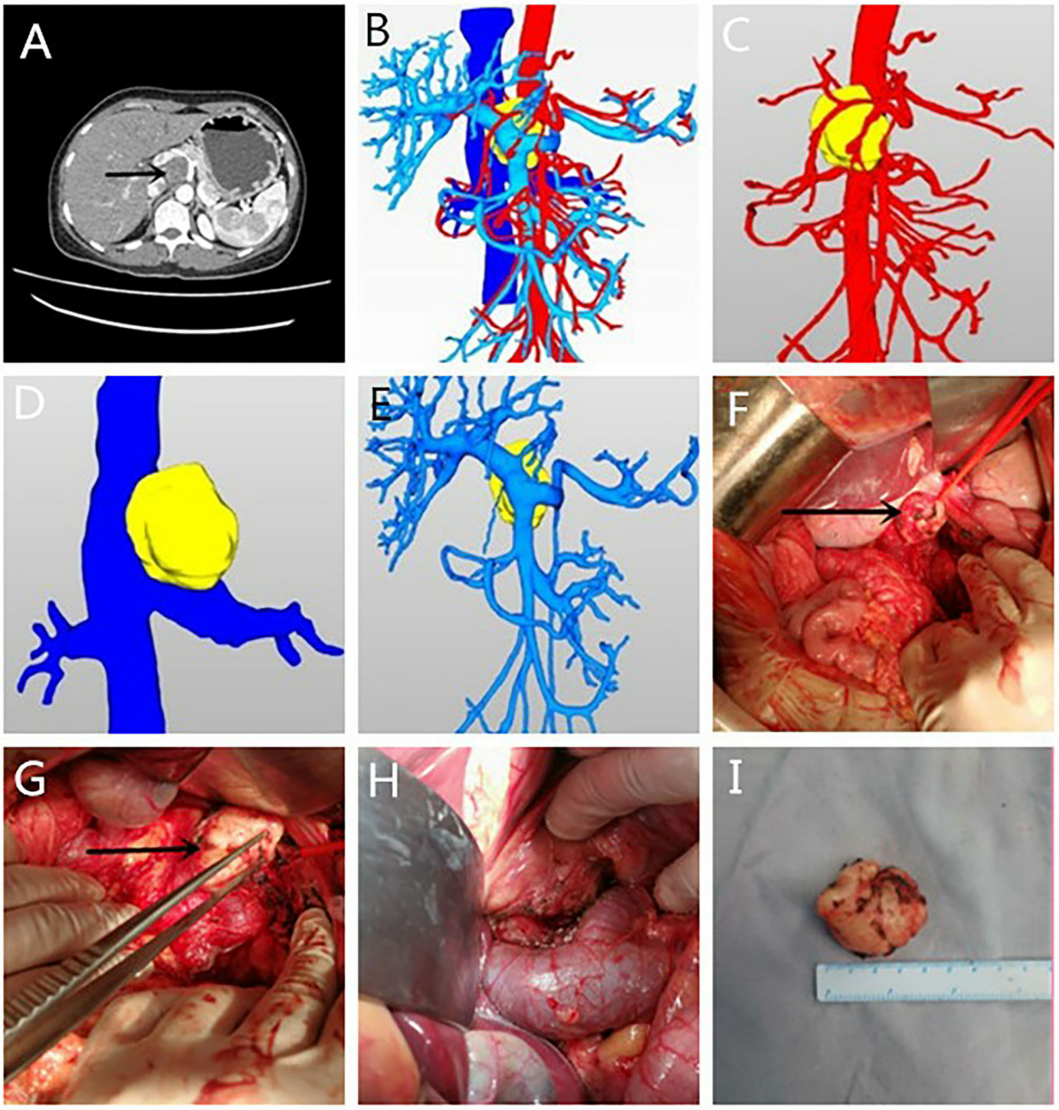
**(A–I)** The enhanced Computed Tomography (CT) and three-dimensional reconstruction performance and the intraoperative situation of case 3.

A 41-year-old woman had pain in right upper abdomen for 1 week. There was no abnormality in colonoscopy at the local hospital and CT considered lymph node metastasis. Enhanced CT after admission revealed (Figure 3) a type of round soft tissue density lesion and a maximum section of about 4.1 cm × 3.8 cm. Three-dimensional reconstruction clearly showed the confluence of the splenic vein and superior mesenteric vein, main portal vein, common hepatic artery, and pancreas were anterior to the tumor; the main abdominal aorta was posterior to the left; the main inferior vena cava was posterior to the right; the celiac trunk was superior to the left; the splenic vein and pancreas were middle to the left; the main superior mesenteric artery was inferior to the left; the main portal vein and proper hepatic artery were inferior to the right; the superior mesenteric vein was anterior to the inferior; the left renal vein was posterior to the inferior; the coronary vein and liver were superior to the left. This patient was taken a reverse “L” incision in the upper right abdomen. The mass was posterior to the head and neck of the pancreas, and was locally surrounded by the common hepatic artery and splenic vein, which was hard and solid, with an intact capsule. The loose tissue between the tumor and the vein was separated and dissected along with the capsule from the right side of the tumor. It was completely dissected along the tumor surface, ligated and cut into the tumor vascular lymphatic tract tissue, and the specimen was removed en bloc. The operation was uneventful, with intraoperative bleeding of about 50 ml (Figures 3F–H). The postoperative histological report suggested retroperitoneal schwannoma with abundant local cells. Immunohistochemical staining was positive for S-100 and SOX10, negative for CD34, CD117, Dog-1 and CD57, and ki-67+ (1–2%).

## Discussion

Retroperitoneal schwannomas usually occur in patients between 40 and 60 years of age and are more common in women than in men (11). However, their age of onset may be younger when immediately adjacent to vital vessels, and this difference may reflect a larger proportion of patients with symptoms in our study population who usually develop symptoms early enough to be diagnosed at a younger age. Tumors are usually asymptomatic when they are small and can be detected inadvertently on imaging studies. The onset of symptoms is due to the compression of adjacent structures by large tumors. Therefore, tumors can grow slowly and are often found to be large, and the symptoms are correspondingly different according to the structures involved. It is worth noting that when the retroperitoneal tumor is located on the waist, even if the tumor is <5 cm, it is more likely to cause hydronephrosis (12). However, our study showed that when the tumor was immediately adjacent to the blood vessels, patients were symptomatic at the time of initial diagnosis, and abdominal pain discomfort was predominant, even when the tumor was small. This may be because compression of blood vessels and nerve irritation can occur in the early stages of the tumor. But the symptoms of these tumors are non-specific and are usually caused by the effects of the tumor on adjacent structures, so it depends on the location and size of the tumor (13). However, neurological symptoms are rare (5). The retroperitoneal position is related to its larger size, which leads to a close relationship with the abdominal large blood vessels and strong adhesion (5). Surgeons need to be familiar with and master surgical techniques.

Imaging examination is a necessary condition for evaluation of surgery and planned treatment. CT and MRI are currently the most commonly used detection methods and provide preoperative visualization. The radiological features are often ambiguous. Therefore, preoperative diagnosis of retroperitoneal schwannoma is very difficult regardless of the use of advanced imaging techniques, and biopsy is not recommended due to the increased risk of complications (14). Especially when complex blood vessels are adjacent, the risk of blood vessel damage is greater. At the same time, due to the heterogeneity within the tumor lesions, the needle biopsy may be inaccurate (15). Because the cell pleomorphism in the degenerated area may lead to the misdiagnosis of malignant tumors. If nuclear atypia, mitotic figures, and vascular invasion are observed histologically, the diagnosis of malignant retroperitoneal schwannoma can be suggested (16).

It is difficult to correctly diagnose schwannoma and distinguish between benign and malignant through imaging analysis, and needle biopsy has certain limitations. Therefore, complete surgical resection is recommended in almost all cases (17) and allows an accurate pathological examination. Surgical resection is the most effective treatment. This method can fully relieve the symptoms caused by the space-occupying effect. When the tumor is attached to the main blood vessels, the risk of excessive bleeding and adjacent organ injury increases. Therefore, adequate preparation of blood before surgery, multi-disciplinary consultations such as vascular surgery, and urology if necessary to deal with possible accidents. When adrenal pheochromocytoma cannot be excluded, fluid expansion preparation should be performed before surgery. The intraoperative frozen section is helpful to determine the resection margin and is essential to determine the extent of surgical treatment, especially for malignant tumors. Local recurrence and overall survival are closely related to negative resection margin and pathological type (18). The surgical approach should be individualized and discussed according to the characteristics of the lesion, the involvement of the vessels, and the experience of the surgeon. Most authors believe that to confirm the diagnosis and rule out any possible malignancy, curative resection should be performed to obtain a negative soft tissue margin, even including the sacrifice of surrounding structures, as incomplete resection results in local recurrence in ~10–20% of patients (19). However, the size and regional anatomy of the schwannoma pose significant difficulties in surgical management. The retroperitoneum is deeply located, has a narrow space, and is inconvenient to perform, which may require an extended incision and continuous use of vascular traction bands to fully expose the tumor and protect adjacent vital structures. Besides, schwannomas can be tightly adherent to adjacent vital vessels (2, 9) or organs (20), and some patients require additional visceral resection.

The relationship between tumor and blood vessels and kidney makes it impossible to accurately distinguish whether there is invasion before surgery, while the three-dimensional reconstruction model can rotate, zoom, and combine display in any dimension, and provides the overall concept, avoiding that clinicians completely rely on subjective imagination for judgment, clarifying the vascular relationship of the tumor, etc., so that the surgeon can predict which blood vessels the specific steps may encounter and prevent injury, find the potential gap, guide the selection of surgical pathway, and reduce the difficulty of surgery and the occurrence of complications. This study showed that the tumor had no significant invasion of peripheral blood vessels, the tumor volume was accurately measured and virtual resection was performed, the loose tissue between the tumor and blood vessels was selected by the surgical approach, the blood vessels were dissected and pulled, the preoperative planning was perfect, and the operation was performed as planned during the operation. For retroperitoneal schwannoma originating from the blood vessel wall, part of the vessel wall may have to be removed and a vascular prosthesis reconstructed (2), which is crucial to consider preoperatively. Therefore, for retroperitoneal masses adjacent to major vessels, schwannomas arising from the great vessels should be used as a differential diagnosis. If the malignant tumor invades major blood vessels, preoperative vascular embolization may help reduce blood loss and prevent uncontrollable bleeding (21). Since it originates from nerves, care must be taken to protect the nerves during surgery. During the slow growth of the tumor, due to the functional compensation of the adjacent spinal nerve roots, complete neurological deficits after the complete sacrifice of the affected spinal nerve roots are rare (20, 22), and the incidence is about 23% (20). Studies have also shown that leaving a part of the capsule next to the nerve can avoid postoperative neurological deficits, which is beneficial to the prognosis (23). Besides, the use of continuous Electromyogram (EMG) monitoring and stimulation can achieve the greatest degree of safe resection, and in some cases can improve the range of resection (7). Uribe et al. found that the use of neuromonitoring can reduce the incidence of complications to <1% (8). In this study, complete tumor resection was performed without neurological complications. Based on previous experience, it is recommended that a complete tumor resection should be performed. Neural monitoring can be used in units with conditions.

Laparoscopic resection of large retroperitoneal schwannomas, even if they are attached to important blood vessels, is feasible and safe under the operation of experienced surgeons (9, 10). But it is more difficult and challenging than open surgery. A certain amount of adipose tissue on the surface of the tumor should be preserved during the operation because it helps to retract, grasp, and remove the tumor. For pheochromocytoma, this grabbing method reduces direct tumor contact, thereby avoiding blood pressure fluctuations (24). For retroperitoneal schwannomas located in the pelvis or close to the ureter, implanting a double J tube into the ureter through a cystoscope may help protect it from damage (25). We emphasize that the appropriate surgical approach should be selected in combination with the characteristics of their cases. This group of patients underwent laparotomy with satisfactory results. Open surgery should be preferred in recurrent schwannomas as well as in those with tumor rupture, hemorrhage, and suspicion of malignancy (26).

The prognosis of this disease is good, and postoperative complications are rare, the most common is recurrence (5–10%), which is related to the rupture or incomplete resection of the tumor (26). The importance of complete resection is, therefore, emphasized (27). Because of its insensitivity to radiation and chemotherapy, re-surgical resection is recommended for recurrent schwannoma (28). Malignant schwannomas have a poor prognosis and a high recurrence rate, so early diagnosis is important while emphasizing the necessity of close postoperative follow-up (29). We should know that regardless of the nature of the tumor, the difficulty of surgery is closely related to the size of the mass and adjacent adhesions, so the importance of early surgery is emphasized instead of only considering the surgical technique (30). Our study confirmed that benign tumors with large size and adjacent complex vessels can still be completely resected with a satisfactory prognosis. We know that the number of cases in this study is small, but we summarized the possible clinical characteristics and surgical strategy in combination with the relevant literature, which can provide clinicians with relevant diagnostic and therapeutic references and has certain clinical significance.

## Data Availability Statement

The original contributions presented in the study are included in the article/supplementary materials, further inquiries can be directed to the corresponding author.

## Ethics Statement

The studies involving human participants were reviewed and approved by Provincial Hospital Affiliated to Shandong University Biomedical Research Ethics Committee. The patients/participants provided their written informed consent to participate in this study. Written informed consent was obtained from the individual(s) for the publication of any potentially identifiable images or data included in this article.

## Author Contributions

QW was responsible for project design, data analysis, and paper writing. BL collected data. JL participated in revising the paper. HC was responsible for drawing up writing ideas, guiding writing articles, and finalizing the draft. All authors contributed to the article and approved the submitted version.

## Conflict of Interest

The authors declare that the research was conducted in the absence of any commercial or financial relationships that could be construed as a potential conflict of interest.
